# Major Revisions in Arthropod Phylogeny Through Improved Supermatrix, With Support for Two Possible Waves of Land Invasion by Chelicerates

**DOI:** 10.1177/1176934320903735

**Published:** 2020-02-05

**Authors:** Katherine E Noah, Jiasheng Hao, Luyan Li, Xiaoyan Sun, Brian Foley, Qun Yang, Xuhua Xia

**Affiliations:** 1 Department of Biology, University of Ottawa, Ottawa, ON, Canada; 2 College of Life Sciences, Anhui Normal University, Wuhu, China; 3 Nanjing Institute of Geology and Paleontology, Chinese Academy of Sciences, Nanjing, China; 4 Theoretical Biology and Biophysics Group, Los Alamos National Laboratory, Los Alamos, NM, USA; 5 Ottawa Institute of Systems Biology, University of Ottawa, Ottawa, ON, Canada

**Keywords:** Deep phylogeny, alignment method, codon degeneration method, arthropods, land colonization

## Abstract

Deep phylogeny involving arthropod lineages is difficult to recover because the erosion of phylogenetic signals over time leads to unreliable multiple sequence alignment (MSA) and subsequent phylogenetic reconstruction. One way to alleviate the problem is to assemble a large number of gene sequences to compensate for the weakness in each individual gene. Such an approach has led to many robustly supported but contradictory phylogenies. A close examination shows that the supermatrix approach often suffers from two shortcomings. The first is that MSA is rarely checked for reliability and, as will be illustrated, can be poor. The second is that, to alleviate the problem of homoplasy at the third codon position of protein-coding genes due to convergent evolution of nucleotide frequencies, phylogeneticists may remove or degenerate the third codon position but may do it improperly and introduce new biases. We performed extensive reanalysis of one of such “big data” sets to highlight these two problems, and demonstrated the power and benefits of correcting or alleviating these problems. Our results support a new group with Xiphosura and Arachnopulmonata (Tetrapulmonata + Scorpiones) as sister taxa. This favors a new hypothesis in which the ancestor of Xiphosura and the extinct Eurypterida (sea scorpions, of which many later forms lived in brackish or freshwater) returned to the sea after the initial chelicerate invasion of land. Our phylogeny is supported even with the original data but processed with a new “principled” codon degeneration. We also show that removing the 1673 codon sites with both AGN and UCN codons (encoding serine) in our alignment can partially reconcile discrepancies between nucleotide-based and AA-based tree, partly because two sequences, one with AGN and the other with UCN, would be identical at the amino acid level but quite different at the nucleotide level.

## Introduction

Arthropod phylogeny has been controversial. The deep divergence can lead to equally supported trees leading to phylogenetic distortion^1,2^ and rapid radiation in some lineages such as Arachnida results in short branch lengths that are difficult to resolve.^3,4^ However, much of the controversy is due to diverse types of data that have been used, including molecular data ranging from rRNA to protein-coding genes from nuclear or mitochondrial source, and morphological characters based on fossils or on various developmental stages of extant organisms.^5^ Most recent phylogenetic effort on arthropods has been concentrated on taking the supermatrix approach, by concatenating either rRNA genes,^6,7^ mitochondrial,^8-11^ or nuclear protein-coding genes.^3,12-25^ This approach is justified because the erosion of phylogenetic signals over time leads to weak phylogenetic signals in each individual gene so that the shared signals among many genes tend to result in finer resolution of phylogenetic relationships, ie, weak effects demanding large sample size to detect.

The supermatrix approach has brought molecular phylogeneticists to a rather awkward situation, with many “robustly supported,” but contradictory/incompatible arthropod phylogenies as exemplified in various chapters of a recent, beautifully edited book.^26^ The editors wisely offered just one solution to resolve the conflicts, and it is to search for “sources of error” (Wägele and Bartolomaeus,^27^ original emphasis), although the Myriapoda + Hexapoda grouping favored in the paper is no longer tenable given the overwhelming evidence against it (Giribet and Edgecombe^28^ and references therein). Here, we aim to identify two sources of error in the sequence alignment and data manipulation before the actual phylogenetic analysis.

Multiple sequence alignment (MSA) is difficult to obtain with divergent lineages because of erosion of homology over time.^29-35^ A poor alignment typically leads to bias and inaccuracy in phylogenetic estimation.^29,32,34,36^ The problem is aggravated with the necessarily large number of sequences needed to represent all major descending lineages of an ancient ancestor because a large data set often necessitates the use of fast and dirty alignment methods without further manual fine-adjustment. We illustrated this by the MSA from Regier et al^19^ (Supplemental file nature08742-s2.nex) which represents one of the best assembled multi-gene supermatrices. Improving the MSA significantly improves phylogenetic resolution and accuracy.

**Figure 1.**
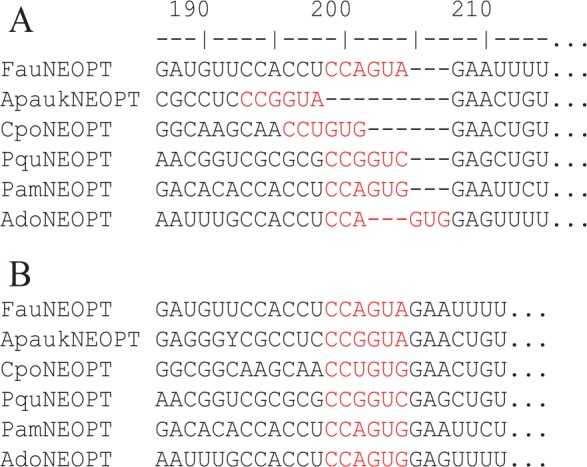
Part of multiple alignment for a subset of six species (A) taken from the Supplemental file (nature08742-s2.nex) in Regier et al.^19^ Re-alignment by MAFFT with options “–localpair −maxiterate 100” is shown in (B). Note that the two codons highlighted in red (coding for amino acids Pro and Val) are identical among the six species. The SP score (a proxy of multiple alignment quality, see text) is 86 for the alignment in (A), but 3591 in (B).

Part of the MSA is shown in Figure 1A, together with an alternative alignment (Figure 1B) obtained by running MAFFT^37^ with optimal settings. Alternatively, one could translate the codon sequences into amino acid sequences, align the amino acid sequences and then map the codon sequences according to the aligned amino acid sequences. This method is implemented in DAMBE since 2000 (Xia,^38(pp38-39)^), with pros and cons illustrated in more detail in Xia.^39(pp72-75)^ This latter approach also results in the alignment in Figure 1B. Although the two MSAs in Figure 1A and B both represent our evolutionary hypotheses, phylogeneticists in general tend to favor the MSA in Figure 1B over MSA Figure 1A. For example, if we use the sum-of-pairs (SP) criterion^40-44^ implemented in DAMBE for evaluating MSA, we get only 86 for the alignment in Figure 1A, but 3591 for the alignment in Figure 1B (A larger SP score means a better MSA). Other alignment problems in the MSA from Regier et al^19^ that may distort phylogenetic signals were illustrated in Supplemental file S1.docx, with the original MSA and improved MSA contrasted in Supplemental Figures S1 to S3.

The second source of error comes from various ways of highlighting phylogenetic signal by noise reduction and filtering. For example, homoplasy at the third codon position of protein-coding genes occurs often due to convergent evolution of nucleotide frequencies of which dramatic changes could occur within a short period of time.^45^ Genes can switch strands and different strands can have very different mutation spectra.^46-48^ The sequences from the diverse array of arthropod taxa compiled by Regier et al^19^ do differ much in nucleotide frequencies, with GC content at the third codon position (GC_3_%) varying from 37.88% to 80.42% in the three ostracods and from 24.10% to 64.40% in arachnids.

Regier et al^19^ wisely degenerated the third codon position, eg, A and G to R, and C and U to Y. The benefit may be illustrated with the following example. If homologous sequences A, B, C have UUA, CUU, and UUU at the same site. The first two codons are Leu codons, but the third is a Phe codon. Thus, at this site, sequences A and B are identical at amino acid level, but both differ from sequence C. However, at the nucleotide level, sequences A and B differ by one transition and one transversion. In contrast, sequences B and C, albeit having two nonsynonymous codons, differ by only a single transition. Thus, a nucleotide-based model would find sequences B and C closer than sequences A and B, while amino acid sequences will group sequences A and B (identical in amino acid Leu) to the exclusion of sequence C (having amino acid Phe). Regier et al^19^ would degenerate the three codons in the three sequences to YUN, YUN, and UUY, respectively, so sequences A and B are now identical and both differ somewhat from sequence C, consistent with the amino acid sequences. The degenerated sequences generate phylogenetic trees much more robust and meaningful than undegenerated sequences or amino acid sequences.^19^

**Figure 2.**
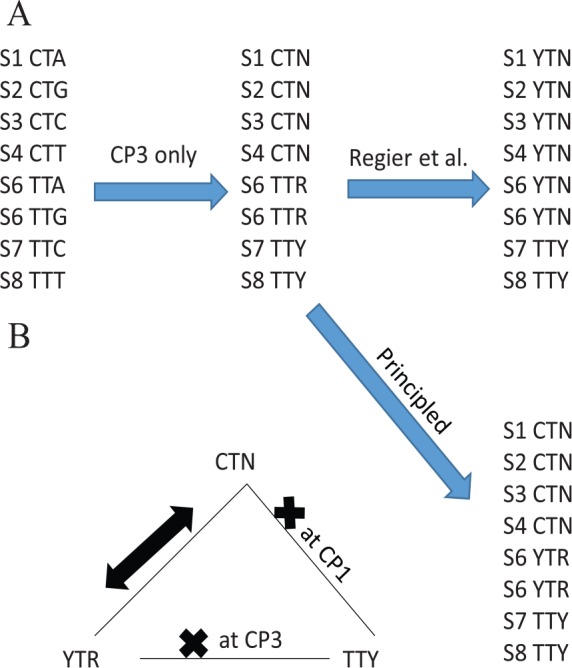
Contrasting “principled” protocol of degenerating codons in compound codon families with that in Regier et al, with the principle that degenerated synonymous codons are “compatible” with each other (ie, having at least one shared codon between them) and consequently will replace each other more frequently than with nonsynonymous codons (A). This “principled” degeneration is also important for nucleotide-based analysis (B), illustrated with differential alignment scores between the two contrasting protocols of degeneration. A heavy “×” means incompatible.

However, there are some problems in the codon degeneration in Regier et al.^19^ The principle of degenerating codons is to (1) make synonymous codons “compatible” so that synonymous codons can substitute into each other with a higher rate than that between nonsynonymous codons and (2) avoid losing too much phylogenetically useful information through codon degeneration. The degenerated Leu codons YTR and CTN are compatible because they represent two sets of codons with shared codons. For example, CTA and CTG are present in both sets of codons represented by YTR and CTN and serve as an evolutionary path linking the two sets of synonymous codons. In contrast, two sets of nonsynonymous codons, such as that represented by TTY (coding Phe) and CTN (coding Leu), should not be “compatible.” That is, they should have no shared codons between them. However, the degeneracy protocol taken by Regier et al^19^ violated this principle (Figure 2) by further degenerating CTN and TTR codons all to YTN codons. YTN is heterogeneous and include both Leu and Phe codons. Such degeneration obscures the difference between Phe and Leu codons. Phe and Leu differ much in side chain and should not be lumped together. For example, Miyata’s distance between Phe and Leu is 0.63.^49^ According to Figure 13.1 in Xia,^50^ such an amino acid dissimilarity would reduce amino acid replacement by 41% relative to synonymous substitutions (with amino acid dissimilarity 0).

We propose a “principled” coding scheme (Figure 2A) which would degenerate Leu as CTN and YTR, with the operational principle being that, for two synonymous codon subfamilies of different sizes (eg, one with four codons and the other two), we degenerate codon positions 1 or 2 only in the small codon subfamily. For example, CTN has four codons and TTR has two codons (ie, the smaller of the two), so we degenerate the first codon position of only the smaller TTR family to YTR (Figure 2A). This ensures that synonymous codons CTN and YTR are compatible with each other but not with TTY (Figure 2B).

This “principled” degeneracy protocol can be applied to any codon family with two unequal sized subfamilies. In addition to the 6-codon family with 4-codon and 2-codon subfamilies illustrated in Figure 2A, the method can also be applied to the Lys codon family coded by AAA, AAG, and AGG in genetic code 24. Here, we have a subfamily with two synonymous codons (AAA and AAG) and a smaller subfamily with only one AGG codon. According to the “principled” degeneration, AAA and AAG can be degenerated to AAR, and AGG degenerated to ARG. Note that we degenerate the second codon position only for the smaller codon subfamily. AAR and ARG are compatible with each other but not compatible with AGA that is a Ser codon in genetic code 24. It would be inappropriate to degenerate the three codons into ARR that would have included the Ser codon AGA.

**Figure 3.**
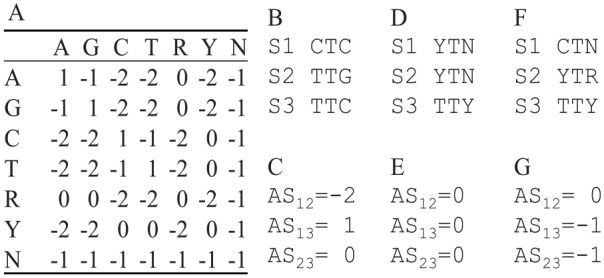
Effect of different codon degeneration methods on pairwise alignment score. (A) Match/mismatch score matrix for computing pairwise alignment score (AS). Matrix entries involving ambiguous codes are mean values, eg, the entry for A/R is the average between A/A and A/G. (B) Three aligned sequences (S1, S2, and S3) each containing just one codon. S1 and S2 encode a Leu codon (CTC and TTG, respectively), and S3 encodes a Phe codon. (C) Three pairwise alignment scores for sequences in (B), with AS_12_ between S1 and S2, AS_13_ between S1 and S3, and AS_23_ between S2 and S3). (D) S1 to S3 after codon degeneration following Regier et al^19^ and the associated pairwise alignment scores (AS_12_, AS_13_ and AS_23_) in (E). (F) S1 to S3 after the “principled” codon degeneration and the associated pairwise alignment scores (AS_12_, AS_13_ and AS_23_) in (G).

The “principled” degeneracy protocol is important not only for codon-based analysis, but also for nucleotide-based analysis, ie, with nucleotide-based substitution models. This is illustrated with alignment scores in Figure 3, but can be equally well illustrated with phylogenetic distances. Given two aligned sequences, a matched nucleotide site will gain one point (match score = 1), a site with a transitional difference is penalized with a mismatch score of −1, and a site with a transversional difference has a penalty of −2 (Figure 3A). A site with A/R (where R stands for purine) implies either an A/A match with a score of 1 or an A/G mismatch with a score of −1, so the matrix entry for A/R is (1 − 1)/2 = 0 (Figure 3A). An A/Y site is always a transversion, hence a score of −2 (Figure 3A).

Given the match/mismatch score matrix (Figure 3A) and a set of aligned codon sequences, we can compute alignment score (AS, which often serve as a proxy of homology) between each pair of sequences. For illustration, suppose we have three sequences each containing just a single codon (S1 to S3, Figure 3B). S1 (=“CTC”) and S2 (=“TTG”) encode a Leu codon, and S3 (=“TTC”) encodes a Phe codon. Site-by-site comparison between S1 and S2 yields a transitional difference (with a score of −1) at site 1, one match (with a score of 1) at site 2, and one transversion (with a score of −2) at site 3, so the alignment score between S1 and S2 (AS_12_) is (-1) + 1 + (-2) = –2 (Figure 3 C). Thus, we have AS_12_ = –2, AS_13_ = 1, and AS_23_ = 0 (Figure 3 C). This is not desirable because AS is smaller between two synonymous Leu codons (S1 and S2) than that between two nonsynonymous codons (between S1 and S3, and between S2 and S3).

The awkward problem above is alleviated with the codon degeneration method used in Regier et al^19^ shown in Figure 3D, which leads to AS_12_ = AS_13_ = AS_23_ = 0 (Figure 3E). The only remaining problem is that the codon degeneration is overdone and obscured the difference between nonsynonymous codons, ie, AS_12_ (between two synonymous codons) becomes the same as AS_13_ and AS_23_ (both between two nonsynonymous codons). The “principled” protocol (Figure 3 F) of codon degeneration gives us AS_12_ = 0, AS_13_ = AS_23_ = –1 (Figure 3G), which reflects our understanding that homology between two synonymous codons should be greater than that between two nonsynonymous codons.

We performed extensive reanalysis of the data in Regier et al^19^ by improving sequence alignment and codon degeneration. This resulted in increased phylogenetic resolution of deep nodes. In particular, our results support a new group with Xiphosura and Arachnopulmonata (with Scorpiones and Tetrapulmonata) as sister taxa which is consistent with embryological evidence^51^ and several recent publications.^3,52^

## Materials and Methods

Regier et al^19^ includes three supplemental files with sequence alignment from 62 concatenated protein-coding genes (68 gene regions) and 80 taxa, with gene boundaries between gene regions demarcated by “NNNNNN.” The file nature08742-s2.nex is the aligned codon sequences. It is (1) codon-degenerated to produce nature08742-s3Degen1.nex and (2) translated into amino acid sequences and cleaned by removing unalignable segments to produce nature08742-s4AA.nex. Our re-analysis is based on file nature08742-s2.nex.

### Sequence alignment and “principled” codon degeneration

We improved sequence alignment in two ways. The first is to re-align sequences for each of the 68 gene regions with the most accurate options in MAFFT^53^ and MUSCLE.^54,55^ These two programs produce a better MSA than Clustal.^56^ The LINSI option that generates the most accurate alignment (“–localpair” and “–maxiterate = 1000”) is used for MAFFT. For MUSCLE, the default option includes all optimizations and is the slowest and most accurate. The original sequence, after removing all gaps, were first translated into amino acid sequences and aligned by MAFFT/MUSCLE. Codon sequences were then aligned against the aligned amino acid sequences.

We evaluated MSA from MAFFT and MUSCLE by the SP criterion^40-44^ without penalizing shared gaps (SP criterion is simply the sum of all pairwise alignment scores given gap-open and gap-extension penalty and a match/mismatch score matrix). The evaluation of MSA by the SP criterion is implemented in DAMBE (Xia,^57,58^ under menu item “Alignment|Evaluate a multiple alignment”). This resulted in 68 MSA files with the highest SP scores.

Of the 68 sets of homologous gene regions aligned separately by MAFFT and MUSCLE, 26 sets have SP scores higher for the MAFFT alignment than for the MUSCLE alignment, 13 sets show the opposite, and 29 sets have SP scores identical between MAFFT and MUSCLE alignments. The final concatenated sequences (Supplemental file SuperMat.PHY) are from the sets with the highest SP scores, regardless whether it is MAFFT or MUSCLE alignment. There are 27 sets of sequences with the original MSA as good as, or slightly better than, the MAFFT or MUSCLE alignments. The improvement in SP score is shown in Supplemental Figure S4.

**Figure 4.**
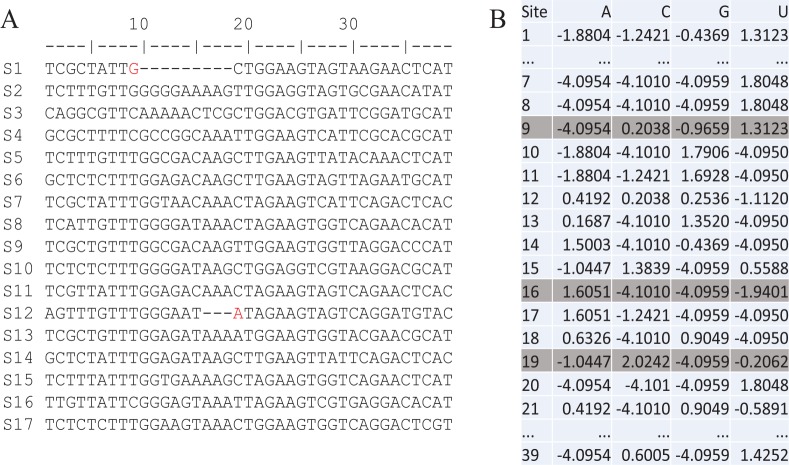
(A) Refine multiple sequence alignment (left panel, with 39 aligned sites) by (B) position weight matrix (right panel, with 39 rows corresponding to 39 sites). Columns aligned sequences with identical nucleotides are indicated by “*.”

The second way of improving MSA is to automatically refine MSA by a position weight matrix (PWM, Xia^59,60^), illustrated with aligned sequences in Figure 4A. PWM is a table of logarithm of the ratio of the site-specific frequency over the background frequency and measures the propensity of a nucleotide or amino acid occurring at a particular site (Figure 4B). A PWM value of zero for nucleotide i at site j means that nucleotide i is neither preferred nor avoided at site j. A value greater or smaller than 0 means that the nucleotide is preferred or avoided, respectively, at site j. The nucleotide G at site 9 has a PWM score of −0.9659 (Figure 4B), ie, G is avoided at this site. In contrast, G is preferred at site 18, with a PWM score of 0.9049 (Figure 4B). Therefore, we should shift the nucleotide G at site 9 in S1 rightward to site 18. Similarly, nucleotide A at site 19 has a PWM score of −1.0447 (Figure 4B), ie, A is avoided at this site. In contrast, A is strongly favored at site 16 with a PWM score of 1.6051, so we should shift the nucleotide A at site 19 in sequence S12 leftward to site 16. This post-alignment refinement with PWM takes little computational time, and is implemented in DAMBE as well (Xia,^57,58^ under menu item “Alignment|Refine sequence alignment”).

After sequence alignment and refinement, the 68 MSA files are concatenated into one supermatrix (Supplemental file SuperMat.PHY). This file can be used to generate the corresponding amino acid sequences or codon-degenerated sequences by using DAMBE.^61^ After reading the sequence file into DAMBE by clicking “File | Open standard sequence file,” click “Sequences | work on amino acid sequences” to generate AA sequences, or click “Sequences | Sequence manipulation | Degenerate synonymous codons” to perform the “principled” codon degeneration.

We have added a few computer utility functions to facilitate the supermatrix approach in phylogenetics. One often has multiple files each containing a set of homologous sequences but different files may have different species although some species are shared among files. One wants to align sequences in each file with optimized options and then concatenate them into a supermatrix for phylogenetic analysis, or analyze sequences in individual files and produce a consensus tree. This can be done with a few clicks in DAMBE (Xia,^58^ although the actual computation time depends on number of species, number of files, and sequence lengths).

### Phylogenetic analysis

We used PhyML^62^ and RAxML^63^ for phylogenetic reconstruction. The GTR +Γ model was used and four discrete rate categories were used for approximate gamma distribution (RAxML always uses four discrete rate categories). For PhyML, the tree improvement option “-s” was set to “BEST” (best of NNI and SPR search). The “-o” option was set to “tlr” which optimizes the topology, the branch lengths and rate parameters. For amino acid sequences, the default “LG” or alternative “JTT” empirical matrix is used. RAxML performs 1000 rapid bootstrap inferences and a thorough ML search.

The codon-degenerated sequences were also analyzed with MrBayes.^64,65^ The GTR +Γ model with a proportion of invariable sites (lset nst = 6, rates = invgamma) is used. We run MCMC for 1,000,000 generations. The other options follow MrBayes default.

## Results

### PhyML tree and RAxML tree are identical in topology and visually indistinguishable in branch lengths

**Figure 5.**
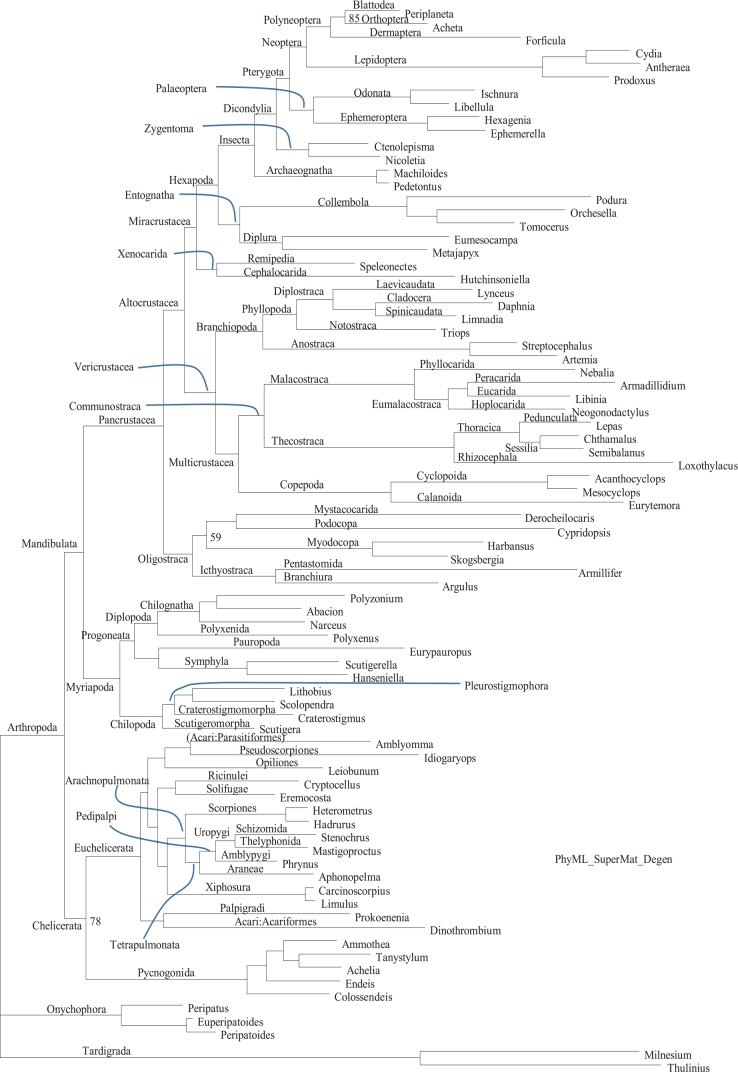
Phylogenetic tree with support values from PhyML based on codon-degenerated data, for comparison with Figure 1 in Regier et al^19^ which is also based on codon-degenerated data. All internal nodes are 100% supported except for two internal nodes with 59% and 78% support (indicated). The sequence name in the leaf nodes are genus names whose matching sequence names are in Appendix 1 of Supplemental file S1.docx. Supplemental Figure S5, from RAxML, shows the same tree with sequence names. Internal node labeling also follows Regier et al.^19^

The phylogenetic tree from PhyML based on codon-degenerated sequences (Figure 5) is visually identical to that from RAxML (Supplemental Figure S5), except for support values which are higher in PhyML than in RAxML. This is expected because PhyML does not do the conventional bootstrapping but used (1-p) as a support value where p is obtained from a quasi-LRT (likelihood ratio test) between the best tree and alternative topologies generated from nearest neighbor interchange.^66^

It is remarkable that the PhyML tree and the RAxML tree are not only identical in topology, but also indistinguishable in branch lengths. While we do expect phylogenetic reconstruction with the same substitution model and the same MSA to generate the same result, in practice such an expectation is rarely realized. RAxML and PhyML use different methods to generate starting trees (RAxML used maximum parsimony and PhyML used BioNJ), and search tree space differently. If phylogenetic signals were weak, then the 80 species could potentially have many similar topologies with similar lnL values, with a high chance of the two programs generating similar but non-identical topologies. In particular, the rate parameters of the substitution model are different, albeit minor, between PhyML and RAxML output (Table 1), yet such estimation noise did not result in PhyML and RAxML arriving at different final topologies.

**Table 1.** Comparison of substitution model (GTR + Γ) parameters and tree statistics between RAxML and PhyML: shape parameter (α) of the gamma distribution, five rate ratio parameters, tree log-likelihood (Tree lnL), and tree size.

	RAxML	PhyML
**α**	0.314998	0.315
**rate A ↔ C**	2.37295	2.41895
**rate A ↔ G**	2.88307	2.93412
**rate A ↔ T**	1.46723	1.49168
**rate C ↔ G**	1.97825	2.01228
**rate C ↔ T**	3.04210	3.09935
**rate G ↔ T**	1	1
**Tree lnL**	-548957	-544342
**Tree size**	7.65749	7.60190

It is even more remarkable that the MrBayes tree (Supplemental Figure S6) also has a topology identical to that from PhyML and RAxML. The node support values are comparable to those of the PhyML tree (Figure 5) and higher than those in the RAxML tree (Supplemental Figure S5). In short, the sequences jointly offer strong phylogenetic signals to resolve arthropod phylogeny.

In order to know assess the effect of improved alignment and the “principled” codon degeneration on the phylogenetic outcome, we have applied the “principled” codon degeneration on the original MSA in Regier et al^19^ and used PhyML for phylogenetic reconstruction with the same options. The resulting topology (Supplemental Figure S7) is again identical to that in Figure 5, but different from that in Regier et al.^19^ Furthermore, the node support values in Supplemental Figure S7 are smaller than those in Figure 5. Thus, both the “principled” codon degeneration and better alignment can have positive impact on phylogenetic resolution.

### Xiphosura is nested within arachnid species

One striking feature in our phylogenetic results is that the two xiphosuran species (*Carcinoscorpius* for *Carcinoscorpius rotundicauda* and *Limulus* for *Limulus polyphemus* in Figure 5) are well nested within arachnid species, in contrast to their position in Figure 1 of Regier et al^19^ where Xiphosura is a sister group to all arachnid species. Relative phylogenetic relationships among the 13 arachnid species are remarkably the same between our Figure 5 and Regier et al’s Figure 1. For example, both recovered Arachnopulmonata (Scorpions + Tetrapulmonata), substantiated not only by orthologous gene sequences,^3,16^ but also by gene/genome duplication events,^52,67,68^ and morphological studies.^69,70^

The main issue here is phylogenetic position of Xiphosura. There are three strong lines of evidence in favor of phylogenetic affinity between Xiphosura and Araneae. Ballesteros and Sharma (2019) performed a thorough phylogenetic study taking into consideration of many potentially confounding factors and integrated phylogenetic studies beyond molecular data. They found Xiphosura nested well within Arachnid species rather than having Xiphosura as an outgroup of Arachnida. The second line of evidence came from studies of gene/genome duplication,^67,68^ ie, a genome duplication in an ancestor will lead to many duplicated genes in all of its descendant lineages. This has helped establishing Arachnopulmonata (Scorpions + Tetrapulmonata) because of their shared sets of duplicated genes. Following these studies, Leite et al^52^ showed that Xiphosura and Arachnopulmonata share sets of duplicated genes, setting them apart from other Chelicerates that do not share this feature of duplicated genes. However, it is possible that the duplicated genes in Xiphosura arose from independent genome duplication or segmental duplication events.^71,72^ The third line of evidence came from embryological studies, with egg morphology, egg composition, and cell division pattern during embryo development most similar between Xiphosura and Araneae,^51^ although the similarity is less obvious with Scorpiones because the latter developed viviparity which results in much smaller eggs and different developmental patterns. However, there are alternative views in support of Xiphosura as an outgroup to Arachnida.

**Figure 6.**
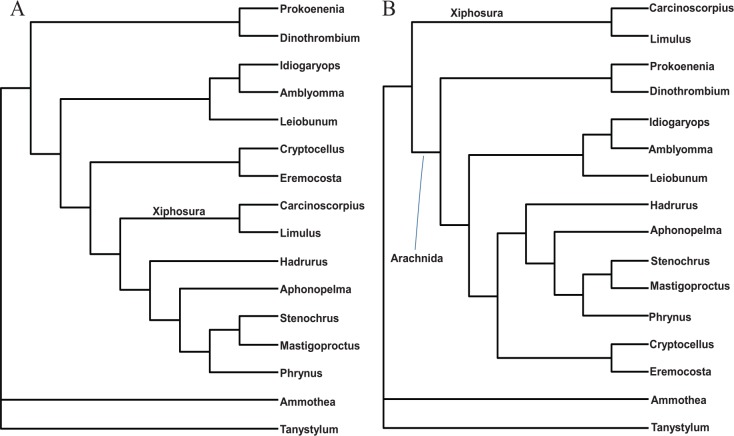
Two alternative topologies for chelicerates, one with Xiphosura nested within arachnid species (A) extracted from Figure 5, and the other with Xiphosura as a sister taxon to arachnid species (B) extracted from Figure 1 in Regier et al.^19^ The two are identical except for the position of Xiphosura. The topology in (B) is strongly rejected by sequence data (*P* < .0001).

We evaluated these two alternative hypotheses depicted in Figure 6A and B. We used a subset of sequences consisting of 20 species in Chelicerata (Euchelicerata + Pycnogonida). Among the 68 aligned gene regions in the data set, gene regions 5, 6, 9, 19, 34, 35, 38, 42, 45, 54, 66, and 68 were shared among Euchelicerata (Arachnida + Xiphosura). We concatenated these sequences to build a supermatrix for chelicerate species, but excluded four species in the sequence file (HspARACH, AeliPYCNO, Col2PYCNO, and ElePYCNO for *Heterometrus spinifer, Achelia echinata, Colossendeis sp.*, and *Endeis laevis*, respectively) which either have missing genes or long stretches of missing sites within a gene.

The resulting Supplemental file (Chelicerate.pml, which includes 12 arachnid species, two xiphosuran species, and two Pycnogonid species) was used to evaluate the two specific alternative phylogenetic hypotheses (Figure 6). The reduced number of species allows us to use a more computation-intensive codon-based model in CODEML in the PAML package.^73^ The resulting lnL is −81953.587 for the topology in Figure 6A, and −82880.871 for the topology in Figure 6B, with standard error of the difference being 130.173. The null hypothesis that the two topologies are equally good is rejected with *P* < 0.0001. Therefore, the phylogenetic results in Regier et al^19^ where Xiphosura is a sister taxon of Arachnida is an artifact, likely due to the alignment or codon-degeneration problems we mentioned before. We applied the “principled” codon degeneration to the original sequence alignment in Regier et al,^19^ and the resulting tree (Supplemental Figure S7) has the same topology as that in Figure 5. Thus, the topological difference in the trees between Figures 1 and 5 in Regier et al^19^ is mainly due to codon degeneration.

The phylogenetic position of Xiphosura and Scorpiones in Figure 5 implies that, after the common ancestor of Euchelicerata had adopted a terrestrial life, the common ancestor of Xiphosura and Scorpiones (and the extinct Paleozoic Eurypterida or sea scorpions) have returned to marine environment. The phylogenetic affinity between Xiphosura and Eurypterida has been well established by paleontologists.^74,75^ “In fact, all recent investigations and discoveries of eurypterids have only served to bring out new homologies of structure between the two groups,” especially in the similarity of compound eyes that are different from mandibulate compound eyes.^74,76^ These findings, together with the phylogenetic relationship in Figure 5 and the phylogenetic affinity between Eurypterida and Scorpiones,^77-79^ suggest that the marine ancestor of Xiphosura and Eurypterida is likely also the ancestor of Arachnopulmonata (= Scorpiones + Tetrapulmonata). Consequently, the Scorpiones lineage (or even all Arachnopulmonata lineage) may have resulted from secondary colonization of land from the marine ancestor. This interpretation is consistent not only with molecular, but also with morphological and paleontological data, as we elaborate below.

First, a Paleozoic fossil scorpion (*Palaeoscorpius devonicus*), interpreted as the most basal member of Scorpiones through an extremely detailed study,^80^ was found in a marine environment with morphological features consistent with marine life (ie, it was not a terrestrial creature swept into a marine environment after death). Subsequent re-examination^81^ suggested that the species may have book lungs but shows a lack of other terrestrial adaptations. Thus, *P. devonicus* is either marine or in a transition to become terrestrial, although the putative book lungs could also be remnants of a terrestrial ancestor. These paleontological observations are consistent with the interpretation, based on Figure 5, that the ancestor of Xiphosura and Scorpiones returned to the marine environment, but a phylogenetic lineage, represented as extant Arachnopulmonata (Scorpiones + Tetrapulmonata) has become secondarily terrestrial. That the scorpion lineage has colonized the land independently has been suggested before.^79^ The dissenting opinion (eg, Legg et al^5^) is further weakened by the sharing of the same set of genes among Xiphosura, Scorpiones, and Tetrapulmonata.^52,67,68^

Second, many similarities exist between scorpions and sea scorpions (Eurypterida),^78,82^ and it is likely that scorpions evolved directly from sea scorpions, although there are arguments against this view.^5^ The view of scorpions evolving from sea scorpions is consistent with the basal scorpion lineage represented by the marine or semi-marine *P. devonicus*.^80,81^ The only difficulty seems to be that the most scorpion-like eurypterids (the mixopteroids) have a telson.^79^ With the grouping of Scorpiones and Xiphosura in Figure 5, the presence of a telson in eurypterids is no longer a problem given that species in Xiphosura also have a well-developed telson. Thus, the presence of a telson is indeed in favor of the hypothesis of Xiphosura and Scorpiones all derived from the same common ancestor.

Traditionally, scorpions were considered primitive arachnids because their similarity to eurypterids.^79^ Our phylogenetic result (Figure 5) shows that Xiphosura, Eurypterida, Scorpiones and Araneae jointly represent a rather derived group within arachnids. Xiphosurans were once considered to be close to the root of arthropods because Xiphosura and Pycnogonida (sea spiders) are both marine and both lack spermatophores.^83^ This has misguided earlier phylogenetic interpretations. For example, Anderson,^51^ assuming that Xiphosura were primitive among arthropods, suggested that Araneae must have some very primitive lineages because their egg morphology, embryonic cell division and early development were nearly identical to those in Xiphosura. In light the phylogenetic evidence in Figure 5 and gene duplication data,^52,67,68^ the lack of spermatophores in Xiphosura and Pycnogonida likely resulted from convergence to a marine life and that spermatophores in the ancestor of eurypterids^83^ and pulmonates were likely acquired independently of those in other arachnids as convergence to a terrestrial life.

Given our molecular evidence in Figure 5 and morphological and paleontological evidence presented above, we would like to revive and revise the traditional Merostomata to include Xiphosura, Eurypterida, Scorpiones and Tetrapulmonata to correspond to the phylogeny in Figure 5. Previous objections to Merostomata, as reviewed in Dunlop et al. (2014), are not strong. The first is that Merostomata was an ecological division instead of a phylogenetic one. However, the phylogenetic affinity between Xiphosura and Scorpiones (Figure 5), the morphological similarity between scorpions and sea scorpions, the basal lineage of scorpions being marine, and their sharing of the same set of duplicated genes are all in favor of grouping them in one taxon. Furthermore, a detailed study of fossilized instars of two eurypterid species revealed many similarities in ontogeny between these eurypterid species and modern Xiphosura.^75^ The clustering together of scorpions and Tetrapulmonata in Figure 5 has also been observed in several other molecular studies using the supermatrix approach.^17,21^ Furthermore, a large number of similarities in book lungs have been observed between scorpions and tetrapulmonate arachnids,^84^ leaving little doubt about the homology of these book lungs in the two groups. Thus, although Scorpiones have evolved viviparity and the associated dramatic reduction in egg size and consequent divergence in develop patterns,^51^ molecular data have recovered its true phylogenetic affinities.

The interpretation above, while largely consistent with existing evidence, is still in need of corroboration. An alternative interpretation is that the ancestor of Xiphosura + Eurypterida represents a lineage separate from the ancestor of Arachnopulmonata.^5^ In that case, there would be no re-colonization of land. That is, the ancestor of Xiphosura + Eurypterida returned to the water, and only representatives of Xiphosura survived to this day.

The close phylogenetic affinity between scorpions and tetrapulmonates (Figure 5) sheds light on morphological observations. A meticulous study of book lungs of scorpions and of tetrapulmonates^84^ identified numerous similarities in their fine structure. However, this valuable finding of undisputable homology in book lungs from scorpions and tetrapulmonates was interpreted as to imply a single origin for the book lungs in a terrestrial arachnid ancestor. Our phylogenetic results (Figure 5) suggest that the book lungs in scorpions and tetrapulmonates may have originated in their common ancestor during the process of re-colonizing the land.

Our phylogeny in Figure 5 also suggests that the loss of appendages from the first opisthosomal segment is not a synapomorphy in arachnids because scorpions have limb buds.^79^ The chilaria on the first opisthosomal segment in Xiphosura are thought to be vestiges of the limbs and may be homologous to the limb buds in scorpions. This suggests that appendages from the first opisthosomal segment may be present in the ancestors of Merostomata, and lost subsequently in Tetrapulmonata. That is, the loss of the appendages in Tetrapulmonata is not inherited from the common ancestor of other arachnids.

While cladistic studies on morphological data have previously grouped Arachnida as a monophyletic taxon, with Xiphosura as a sister taxon,^85,86^ such a phylogenetic pattern has rarely been observed in molecular studies using the supermatrix approach, which typically has Xiphosura nested within Arachnida.^3,17,21,22,87^ Meusemann et al^17^ included only one species (*Acanthoscurria gomesiana*) for Tetrapulmonata, and it is clustered together with one representative species of Xiphosura (*Limulus polyphemus*). This is consistent with Figure 5. In Roeding et al,^21^ which included two species of Xiphosura and seven species of Arachnopulmonata (two scorpion species and five Araneae species), Xiphosura and Arachnopulmonata joined to form a monophyletic taxon just as in Figure 5. The same (Xiphosura + Arachnopulmonata) grouping is also recovered in Sanders and Lee.^22^ Phylogenetic analysis of another comprehensive data compilation^87^ included two arachnopulmonates (a spider *Acanthoscurria gomesiana* and a scorpion *Pandinus imperator*) and one xiphosuran (*Limulus polyphemus*). The two arachnopulmonates are clustered together and form a sister group to Xiphosura (Figure 1 in Von Reumont et al^87^), exactly as shown in Figure 5. The phylogenetic result in Regier et al,^19^ with a monophyletic Xiphosura as a sister taxon to a monophyletic Arachnida, is an exception. Our results from reanalysis of the data in Regier et al^19^ revealed that phylogenetic relationships in this data set concerning Xiphosura and Arachnida are the same as those in Sanders and Lee,^22^ Roeding et al^21^ and Meusemann et al.^17^ Xiphosura was also found to be nested within Arachnida in a recent study with more than a million aligned sites, with Xiphosura clustered with Scorpiones, Pedipalpi, and Araneae, except that Ricinulei was also included in the group.^25^ Regier et al^19^ did acknowledge that their results on Chelicerata were weak.

### Phylogenetic differences between nucleotide-based and AA-based trees

We translated the protein-coding sequences into amino acid (AA) sequences using DAMBE^61^ and analyzed the AA sequences with PhyML with empirical substitution matrices LG or JTT. The tree (Supplemental Figure S8) is consistent to the tree from codon-degenerated sequences (Figure 5) in that both have Xiphosura nested within Araneae. However, this tree exhibits two significant differences in topology from that of codon-degenerated sequences. First, Pycnogonida does not cluster with Euchelicerata to form Chelicerata. Instead, it is a sister group to the rest of Euarthropoda (Supplemental Figure S8). Second, Remipedia and Cephalocarida are widely apart on the phylogenetic tree in contrast to forming a monophyletic Xenocarida as a sister taxon to Hexapoda (Supplemental Figure S8).

The discrepancies between nucleotide-based and AA-based tree have previously been suggested to be at least partially attributable to serine codons encoded by AGY and UCN codon families.^88^ For example, two sequences, one with AGN and the other with UCN, would be identical at the amino acid level but quite different at the nucleotide level.^88^ There are 1673 codon sites with both AGN and UCN codons in our alignment. Removing such codon sites leads to the nucleotide-based phylogenetic tree in Supplemental Figure S9 that are similar to the AA tree (Supplemental Figure S8). This suggests that the AA tree is more likely correct than the nucleotide tree concerning these two particular discrepancies.

## Discussion

**Figure 7.**
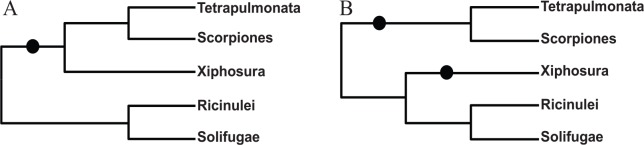
Two alternative phylogenetic positions of Xiphosura. (A) Our tree. (B) Tree from Ballesteros and Sharma (2019). The solid circles indicate genome duplication events.

We need to highlight two uncertainties in our phylogenetic analyses. First, while our phylogenetic result (Figure 5) favors the grouping of Xiphosura and Arachnopulmonata (a taxon including Tetrapulmonata and Scorpiones), with (Ricinulei, Solifugae) as a sister group, such a topology is not significantly different from an alternative topology reported in Ballesteros and Sharma (2019). These two alternative topologies were contrasted in Figure 7. Although it is easy to reject the tree in Regier et al^19^ which has Xiphosura as an outgroup to all other Arachnida, our data cannot reject the two alternative topologies in Figure 7 with any rigorous tests of alternative hypotheses, such as Kishino-Hasegawa test or RELL test.^89^ The (Xiphosura) grouping was previously reported in Roeding et al,^21^ but they did not include representatives of Ricinulei and Solifugae in their phylogenetic analysis. For this reason, the phylogenetic relationship in Figure 7A must be considered as tentative.

Second, there is uncertainty in our interpretation of genome duplication events. We took a parsimony argument and assumed a single genome replication event indicated in Figure 7A. The alternative (eg, Schwager et al^72^) suggests two independent genome duplication events, possibly also involving a slightly different topology (Figure 7B). Unfortunately, the relevant genomes, while being sequenced, are not yet complete. The individual genes that have been used in discriminate between the two scenarios, especially *Hox* genes,^52,67,68,72^ are known to be collinear along the genome and not independent. That is, if one gene in a *Hox* cluster supports one tree, then other genes in the same cluster tend to support the same tree. Thus, genes with one cluster cannot be counted as independent data point supporting either of the two genome-duplication scenarios in Figure 7. It is odd that three horseshoe crab genomes have been reportedly sequenced in 2015 by Kenny et al, but there is still no annotated genome deposited in Genbank—only two horseshoe crab genomes with unannotated contigs are available). We have included a discussion on these alternative possibilities of genome duplication events.

We should also mentioned that the interpretation of *Hox* gene duplication as genome duplication in published papers^52,67,68,72^ on arthropod phylogeny is mainly based on the observation that there is typically only one set of *Hox* genes per genome in invertebrates but often four or eight sets of *Hox* genes in vertebrates.^90,91^ The conventional interpretation of multiple sets of *Hox* genes in vertebrates is that multiple rounds of whole genome duplication in vertebrate lineages lead to multiple sets of *Hox* genes. This interpretation turns out to be not quite correct because, with the availability of many vertebrate genomes, it was found that doubling of *Hox* genes is typically not associated with genome duplication, ie, multiple sets of *Hox* genes in vertebrates are better explained by segmental gene duplication instead of whole genome duplication.^92^ However, segmental gene duplication could still serve as a good phylogenetic marker. That is, genes in the duplicated segment tend to be shared among descendants.

**Figure 8.**
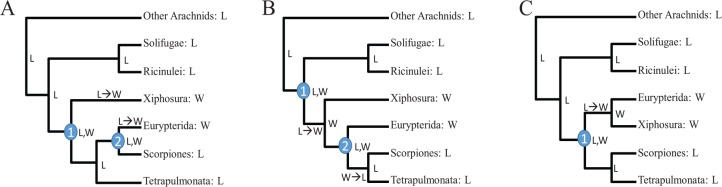
Many alternative evolutionary scenarios of habitat switching (W: water/aquatic; L: land/terrestrial). The three topologies (A, B, and C) are taken from Figure 5, and differ only in the placement of Eurypterida. Ancestral nodes were constructed by Fitch parsimony,^93^ ie, an intersection of the states of the two daughter lineages if the intersection is not empty, but a union of the states of the two daughter lineages if the intersection is empty. Number of union operations represents the minimum number of habitat switches given the tree, eg, (A) and (B) each require at least two habitat switches, and (C) requires just one (more parsimonious than the other two). L→W and W→L indicate possible habitat-switch from terrestrial to aquatic and from aquatic to terrestrial, respectively.

Another uncertainty that we wish to discuss concerns our interpretation of possible re-colonization of land by Scorpiones following the return of their ancestor to the water. There could be many alternative interpretations given the existing evidence (Figure 8). The three topologies in Figure 8 are taken from Figure 5, with Eurypterida added to three alternative positions reflecting uncertainty of phylogenetic position of Eurypterida. Xiphosura and Eurypterida are aquatic (W for water/aquatic), and the rest are terrestrial (L for land/terrestrial). The ancestral nodes are reconstructed with Fitch parsimony.^93^ If Eurypterida is a sister lineage of Scorpiones^77-79^ as shown in Figure 8A, then a minimum of two habitat-switches would be required. Two independent habitat-switches from terrestrial to aquatic (L→W) were hypothesized and indicated in Figure 8A, one along the lineage leading to Xiphosura and another one along the lineage leading to Eurypterida. This interpretation would also imply that the ancestral habitat state for nodes 1 and 2 (Figure 8A) were terrestrial (L), and that the habitat-switches occurred in one of the daughter lineages of these two nodes.

If Eurypterida is a sister lineage to Arachnopulmonata (Scorpiones + Tetrapulmonata), then again a minimum of two habitat-switches is required (Figure 8B). Two independent habitat-switches were indicated in Figure 8B, with one L→W (land/terrestrial to water/aquatic) switch and one W→L switch. However, parsimony reconstruction of ancestral states and state-switches are not unique. For example, if we set all ancestral states to L in Figure 8B, then we again need only two independent L→W switches (one leading to Xiphosura and another to Eurypterida), just as in Figure 1A.

The last topology (Figure 8 C) is the most parsimonious, requiring only one habitat-switch, but it is against the argument that Eurypterida and Scorpiones are closely related.^77-79^ In short, there are two layers of uncertainty, one in the phylogenetic position of Eurypterida and one in the inference of ancestral states given the phylogeny.

In summary, aside from the differences in phylogenetic results highlighted above between ours and those in Regier et al,^19^ our tree in Figure 5 is identical to Figure 1 in Regier et al,^19^ suggesting that their MSA, albeit having some problems as we showed in the introduction, did not lead to serious disruption of phylogenetic relationships. However, our results also suggest that a small effort in data refinement can be well rewarded with increased phylogenetic resolution for some subtrees where phylogenetic signals are weak. The conventional wisdom that researchers have to develop intimacy with their data may go a long way in resolving phylogenetic controversies and reconcile different phylogenetic results. Because published supermatrices are often reused, eg, data in Regier et al^19^ is incorporated in the data of other studies,^87,94^ we hope that our results will alleviate the problem of propagating phylogenetic errors. The data set in Regier et al^19^ is obviously highly valuable, and for this reason it has been reanalyzed in various ways.^95,96^ However, these re-analyses did not improve the data as we did.

## Supplemental Material

S1_xyz309522bb14c8b – Supplemental material for Major Revisions in Arthropod Phylogeny Through Improved Supermatrix, With Support for Two Possible Waves of Land Invasion by Chelicerates Click here for additional data file. Supplemental material, S1_xyz309522bb14c8b for Major Revisions in Arthropod Phylogeny Through Improved Supermatrix, With Support for Two Possible Waves of Land Invasion by Chelicerates by Katherine E Noah, Jiasheng Hao, Luyan Li, Xiaoyan Sun, Brian Foley, Qun Yang and Xuhua Xia in Evolutionary Bioinformatics
