# Supplementary material for: Major Revisions in Arthropod Phylogeny Through Improved Supermatrix, With Support for Two Possible Waves of Land Invasion by Chelicerates
Source: Evol Bioinform Online. 2020 Feb 5;16:1176934320903735. doi: 10.1177/1176934320903735 (PMC7003163; doi:10.1177/1176934320903735)
Supplement: S1_xyz309522bb14c8b – Supplemental material for Major Revisions in Arthropod Phylogeny Through Improved Supermatrix, With Support for Two Possible Waves of Land Invasion by Chelicerates [file S1_xyz309522bb14c8b.pdf]

Supplemental file S1

Katherine E. Noah[1], Jiasheng Hao[2], Luyan Li[3], Xiaoyan Sun[3], Brian Foley[4], Qun Yang[3], Xuhua Xia[15*]

I Other alignment problems in Regier et al. (2010) that may distort phylogenetic signals

Poor alignment creates not only noise but also phylogenetic biases. For example, the original alignment in Regier et al. (2010) in the top part of Fig. S1 increases phylogenetic similarity between the first nine species and the last two species, with the two Archeognatha species (PsaARCHEO for *Pedetontus saltator* and MbaARCHEO for *Machiloides banksi*) and a copepod (A369COPE for *Acanthocyclops vernalis*) being different. However, the last codon in red (Fig. S1) is a lysine codon in all sequences, and the second last is a threonine codon in all but one sequence (A369COPE). The evidence of homology is strong among these codon sites, so they should be aligned as shown in the bottom of Fig. S1.

A similar situation is shown in the top panel of Fig. S2 where the alignment from Regier et al. (2010) introduced an alignment artefact increasing the distance between the first pycnogonid (TorPYCNO for *Tanystylum orbiculare*) and the three other pycnogonid species. The 3-nt deletion in the first sequence (TorPYCNO) is clearly misplaced, with the alignment in the bottom of Fig. S2 having high alignment scores by any reasonable scoring scheme. For example, we may evaluate these two MSA in Fig. S2 by the sum-of-pairs (SP) criterion (Lipman, et al. 1989; Gupta, et al. 1995; Stoye, et al. 1997; Reinert, et al. 2000; Althaus, et al. 2002) without penalizing shared gaps. With match score of 2, transition and transversion penalized by -1 and -2, respectively, and a gap penalty of -3, we obtain SP of 169 for the top MSA, and of 248 for the bottom MSA in Fig. S2. Thus, the bottom MSA is better than the top MSA. In particular, for the top MSA, the alignment score for TorPYCNO and AeliPYCNO is 13 and that for AeliPYCNO and Col2PYCNO is 29, suggesting that AeliPYCNO is more closely related to Col2PYCNO than to TorPYCNO. In contrast, for the bottom MSA, the alignment score for TorPYCNO and AeliPYCNO increases to 45, and that between AeliPYCNO and Col2PYCNO remains unchanged (29, because shared gaps are not penalized), suggesting that AeliPYCNO is more closely to TorPYCNO than to Col2PYCNO, which is consistent with other parts of the MSA.

Because of the high divergence among arthropod sequences, some parts in the MSA were deemed unalignable by Regier et al. (2010) and removed from the translated amino acid

sequences before the final phylogenetic analysis, e.g., the shaded segment in Fig. S3a. This deletion is unnecessary because sequence homology is identifiable as shown in Fig. S3b. Deleting phylogenetically significant signals reduces phylogenetic resolution. However, the deletion of unalignable segments by Regier et al. (2010) is not consistent. While the shaded segment in Fig. S3a is deleted (Regier, et al. 2010, their Supplemental file nature08742-s4AA.nex), the undesirable alignment in Fig. 1a remains in their degenerated sequence file (nature08742-s3Degen1.nex) used to generate their main results in their Figure 1. Thus, their nucleotide sequences and amino acid sequences are not quite comparable. We took the space to show these contrasts because Regier et al. (2010) is not the only paper with sequence alignment problems. Phylogeneticists often implicitly assume that phylogenetic distortion introduced in sequence alignment will be negligible relative to the true phylogenetic signals that remain (which may be true in most cases, but not always).

```
PamNEOPT   AGAACACGAGUUACCAAA---AUGUUGUGCAU
MayEPHEM   AGAUCUCGCGUCACCAAA---AUGUUAUGUCA
EinEPHEM   AGAACCAGAGUUACCAAA---AUUUUAUGUAU
IveODONAT  AGAAGGACUCUCACUAAA---AUGCUUUGUAU
LlyODONAT  CGGAGGAAUAUAACUAAG---AUGCUUUGUUU
StuREMI    AGGAAAAGACUUACCAAA---AUGCUGUGUAU
CliZYGEN   AGGACGAGAGUCACUAAA---AUGCUUUGCAU
NmeZYGEN   AGAUCAAGGGUCACAAAG---AUGUUGUGUAU
JapDIPLUR  AGGACGACAGUGACCAAG---CUCCUGUGCCA
PsaARCHEO  GCCAGAACAAGAGUAACAAAAAUGCUGUGUAU
MbaARCHEO  GCCAGAACGAGAGUAACAAAAAUGUUGUGUAU
A369COPE   AGCGUAACCAGGCGGAGCAAGCUGUUGUGCAA
DtyMYSTACO AGGAGAAGGUGCACCAAA---CUACUCUGUCA
NamDIPLO   AGGAAAAGAUUUACAAAA---UUAUUAUGCCA
```

```
PamNEOPT   ...AGAACACGAGUUACCAAAAUGUUGUGCAU
MayEPHEM   ...AGAUCUCGCGUCACCAAAAUGUUAUGUCA
EinEPHEM   ...AGAACCAGAGUUACCAAAAUUUUAUGUAU
IveODONAT  ...AGAAGGACUCUCACUAAAAUGCUUUGUAU
LlyODONAT  ...CGGAGGAAUAUAACUAAGAUGCUUUGUUU
StuREMI    ...AGGAAAAGACUUACCAAAAUGCUGUGUAU
CliZYGEN   ...AGGACGAGAGUCACUAAAAUGCUUUGCAU
NmeZYGEN   ...AGAUCAAGGGUCACAAAGAUGUUGUGUAU
JapDIPLUR  ...AGGACGACAGUGACCAAGCUCCUGUGCCA
PsaARCHEO  GCCAGAACAAGAGUAACAAAAAUGCUGUGUAU
MbaARCHEO  GCCAGAACGAGAGUAACAAAAAUGUUGUGUAU
A369COPE   AGCGUAACCAGGCGGAGCAAGCUGUUGUGCAA
DtyMYSTACO ...AGGAGAAGGUGCACCAAACUACUCUGUCA
NamDIPLO   ...AGGAAAAGAUUUACAAAAUUAUUAUGCCA
```

Fig. S1. Poor alignment can distort phylogenetic signals. The top alignment, taken from the supplementary file (nature08742-s2.nex) in Regier et al. (2010), confers undue similarity between the first nine and the last two sequences (DtyMYSTACO and NamDIPLO), although the first 12 sequences are phylogenetically more closely related to each other than to the last two sequences. The alternative alignment at bottom have overall higher alignment score and better reflect the true phylogenetic relationship than the alignment at top.

```
TorPYCNO    GCTGTTTTAGGTAAGGTAGCAGCCGAAAAA---TGGGCTGATGTGGTCATTGCT
AeliPYCNO   TCTATAATAGGAAAAGTTTCT---TCTGAAAAATGGGCAGATGTTGTAATTGCA
AhiPYCNO    GCCGTTACCGGAAAGGTTTCT---TCCGATAAGTGGGCAGATGTTGTCATTGCA
Col2PYCNO   GCAATAATTGGTAAGATTCCA---GATAGCAAGTGGAGTGAAGTTGTCCTTGCA

TorPYCNO    GCTGTTTTAGGTAAGGTAGCAGCCGAAAAATGGGCTGATGTGGTCATTGCT
AeliPYCNO   TCTATAATAGGAAAAGTTTCTTCTGAAAAATGGGCAGATGTTGTAATTGCA
AhiPYCNO    GCCGTTACCGGAAAGGTTTCTTCCGATAAGTGGGCAGATGTTGTCATTGCA
Col2PYCNO   GCAATAATTGGTAAGATTCCAGATAGCAAGTGGAGTGAAGTTGTCCTTGCA
```

Fig. S2. Poor alignment at the top, taken from the supplementary file (nature08742-s2.nex) in Regier et al. (2010), unnecessarily increase the distance between TorPYCNO and the three other Pycnogonid species, with the improved alignment at the bottom.

```
(a)                10        20        30        40        50        60
           ----|----|----|----|----|----|----|----|----|----|----|----|--
FauNEOPT   RHASNMGWLNFTFSLQKSFKSLFGEKLEVVRTHQQQENLKFMAHFKRQFVIHQGKRKEILPS
ApaukNEOPT RRAPNMGWLTFTFGLERKFKQLCK-RLEVVRTHQQQETLKFMSHFHRRFIIKDGKRNDKPEG
CpoNEOPT   RRAPNMGWLTFTFGLERKFKQLCK-RLEVVRTHQQQESLKFMSHFHRRFIIRDGKRNQPPEG
PquNEOPT   RHAPNMGWLTFTFGLERKFKSLCT-RLEVVRTHQQQENLKFMAHFNRRFIIKEGKRNGDNKV
PamNEOPT   REASNMGWLTFTFSLQKKFKSLFGEKLEVVRTHQQQENLKFMAHFKRKFIIHQGKRKETLPR
AdoNEOPT   REASNMGWLTFTFSLQKKFKSLFGEKLEVIRTHQQQENLKFMAHFKRKFVIHQGKRKEIPDP
           * * ***** *** *   ** *    *** ******* **** ** * * *  ***

                 70        80        90       100       110       120
           --|----|----|----|----|----|----|----|----|----|----|----|----
FauNEOPT   DVPPPV-EFYHLRSNGSALCTRLIQIRPDASALNSQFCYILKVPLNNQEEEPSGIVYVWIGS
ApaukNEOPT RLPV---ELFELRSNGSALCTRLIQVKADATQLNSAFCYILNVPLEGNSDTSSAIVYAWIGS
CpoNEOPT   GKQPV--ELFELRSNGSALCTRLVQVKADAAQXNSAFCYILNVPLEGANDTSSAIVYAWIGS
PquNEOPT   NGRAPV-ELYELRSNGSALCTRLVQVRADAAQLNSCFCYILNVPLEGADDTXSAIVYVWVGS
PamNEOPT   DTPPPV-EFYHLRSNGSPLCTRLIQIKPDATALNPAFTYILKVPFDNEEQ--SGIVYVWIGS
AdoNEOPT   NLPPP-VEFYHLRSNSSSLCTRLIQIKPDAAALNSAFCYILKVPLNKEEQ--TGIVYVWIGS
                  *   **** * ***** *   **   *  * *** **          *** * **
```

```
(b)
FauNEOPT   LKFMAHFKRQFVIHQGKRKEILPSDVPPPVEFYHLRSNGSALCTRLIQIRPDASALNSQFCY
ApaukNEOPT LKFMSHFHRRFIIKDGKRNDKPE--GRLPVELFELRSNGSALCTRLIQVKADATQLNSAFCY
CpoNEOPT   LKFMSHFHRRFIIRDGKRNQPPE-GGKQPVELFELRSNGSALCTRLVQVKADAAQXNSAFCY
PquNEOPT   LKFMAHFNRRFIIKEGKRNGDNKVNGRAPVELYELRSNGSALCTRLVQVRADAAQLNSCFCY
PamNEOPT   LKFMAHFKRKFIIHQGKRKETLPRDTPPPVEFYHLRSNGSPLCTRLIQIKPDATALNPAFTY
AdoNEOPT   LKFMAHFKRKFVIHQGKRKEIPDPNLPPPVEFYHLRSNSSSLCTRLIQIKPDAAALNSAFCY
           **** ** * * *  ***          ***   **** * ***** *   **   *  * *
```

Fig. S3. Unnecessary deletion of phylogenetically informative data. (a) Partial amino acid sequences translated from the codon sequences in the supplementary file (nature08742-s2.nex) in Regier et al. (2010). The shaded segments, including the amino acid E at labelled site 70, were deemed by Regier et al. (2010) as unalignable and removed in the final amino acid sequence alignment for phylogenetic analysis. (b) Translated from our re-aligned codon sequences. The shaded segments contains phylogenetic information consistent with other corroborative evidence that {FauNEOPT, PamNEOPT, AdoNEOPT} and {ApaukNEOPT, CpoNEOPT, PquNEOPT} belong to separate lineages.

II. Measure the degree of sequence alignment improvement

We realigned the 68 gene segments in Regier et al. (2010) with MAFFT (Katoh, et al. 2009) and MUSCLE (Edgar 2004a, b). These two programs produce a better multiple sequence alignment (MSA) than Clustal (Thompson, et al. 1994). The LINSI option that generates the most accurate alignment ('–localpair' and '–maxiterate = 1000') is used for MAFFT. For MUSCLE, the default option includes all optimizations and is the slowest and most accurate. The original sequence, after removing all gaps, were first translated into amino acid sequences and aligned by MAFFT/MUSCLE. Codon sequences were then aligned against the aligned amino acid sequences using DAMBE (Xia 2018). This protocol of aligning codon sequences against amino acid sequences is available since 2000 (Xia 2000).

Multiple sequence alignment (MSA) quality is often measured by the sum-of-pairs (SP) criterion, i.e., the sum of all pairwise alignment scores (without counting shared gaps). For each of the 68 gene segments, we computed SP for the original MSA (SP_{Regier}) and for the new MSA realigned with MAFFT (SP_{MAFFT}) and MUSCLE (SP_{MUSCLE}). For each of the 68 gene segments, we choose the MSA with the highest SP score for final assembly into a supermatrix.

Fig. S4 plots $Max(SP_{MAFFT}, SP_{MUSCLE}) – SP_{Regier}$ for each gene segment. For some gene segments, the realigned MSA is the same as the original so that SP scores for the three sets are identical.

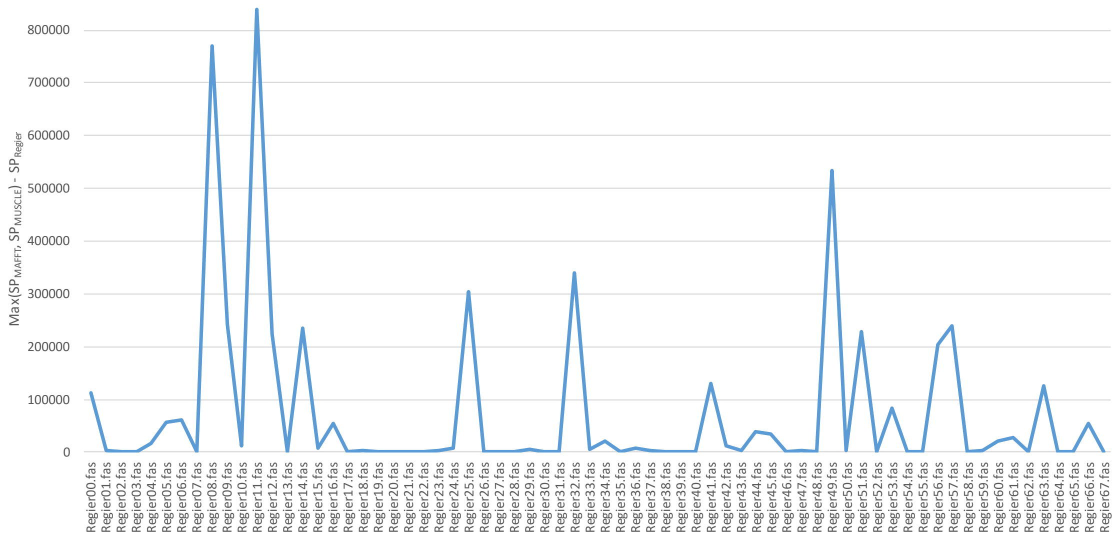

Fig. S4. The improvement of multiple sequence alignment, indicated by the increased SP score, after realigning sequences with the most accurate option in MAFFT and MUSCLE. SP is generated with default options in DAMBE (Xia 2018).

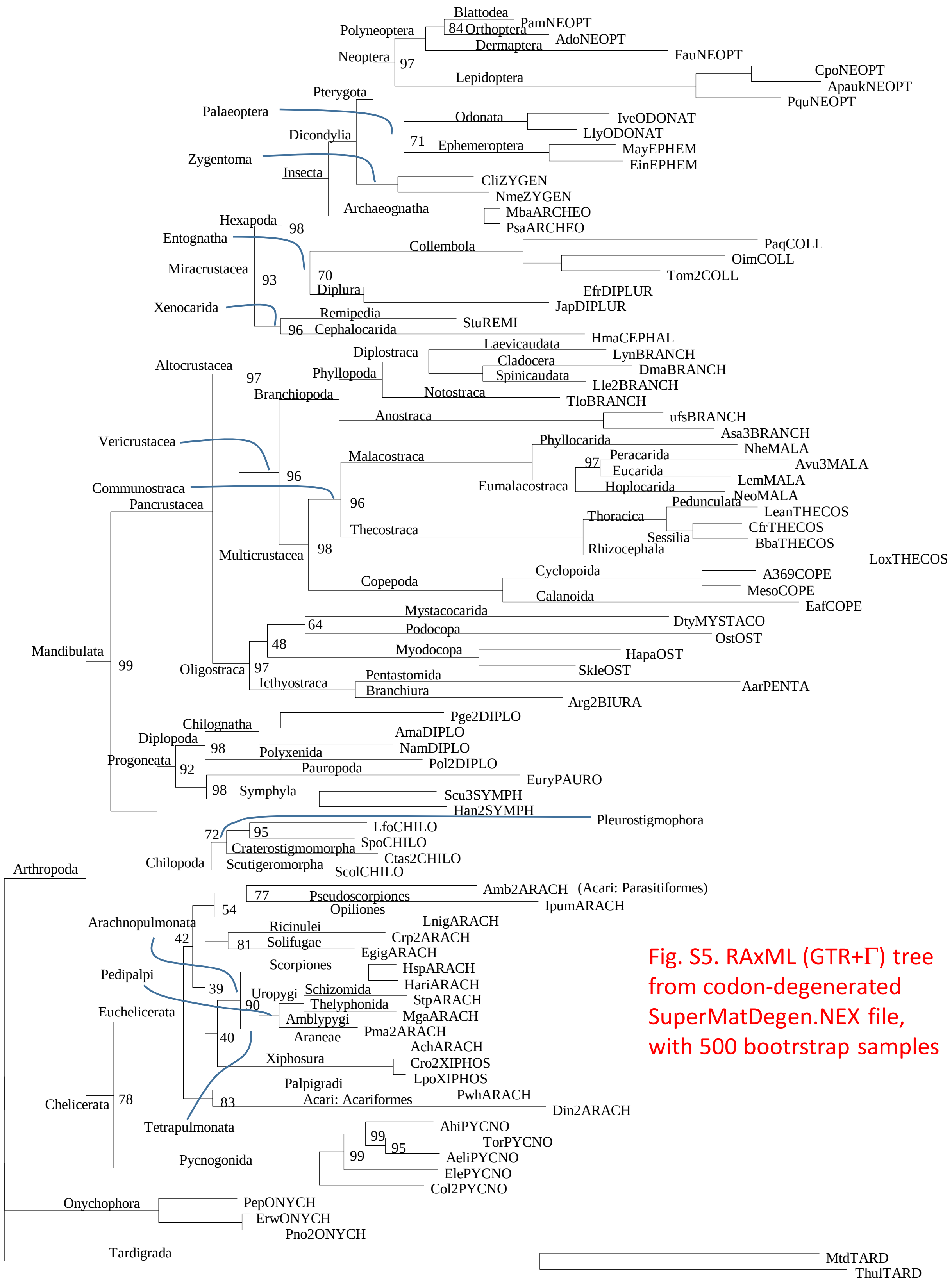

Fig. S5. RAxML (GTR+Γ) tree from codon-degenerated SuperMatDegen.NEX file, with 500 bootrstrap samples

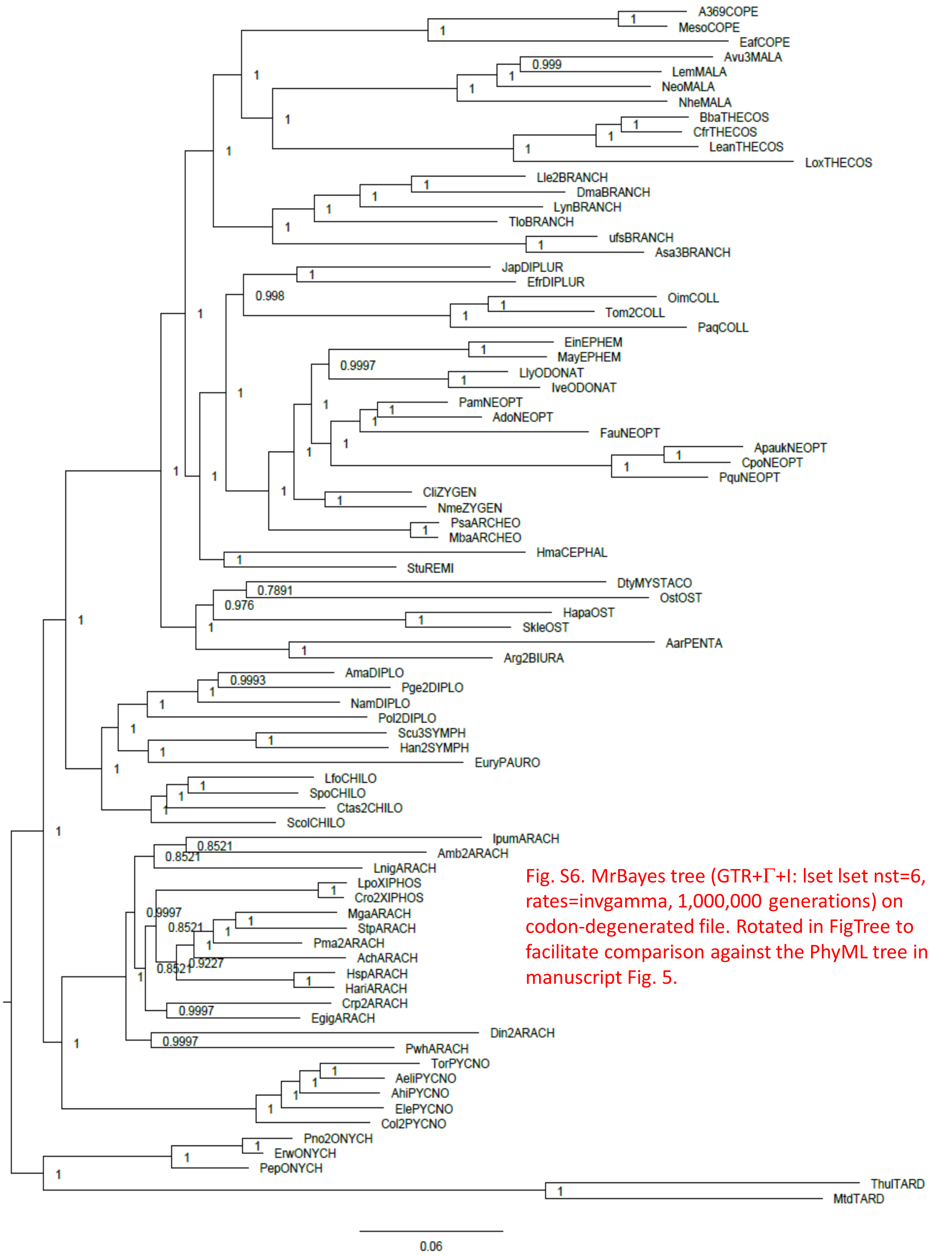

Fig. S6. MrBayes tree (GTR+Γ+I: lset lset nst=6, rates=invgamma, 1,000,000 generations) on codon-degenerated file. Rotated in FigTree to facilitate comparison against the PhyML tree in manuscript Fig. 5.

Fig. S7. PhyML (GTR+Γ) tree from the original MSA in Regier et al. (2010), but codon-degenerated with the "principled" method

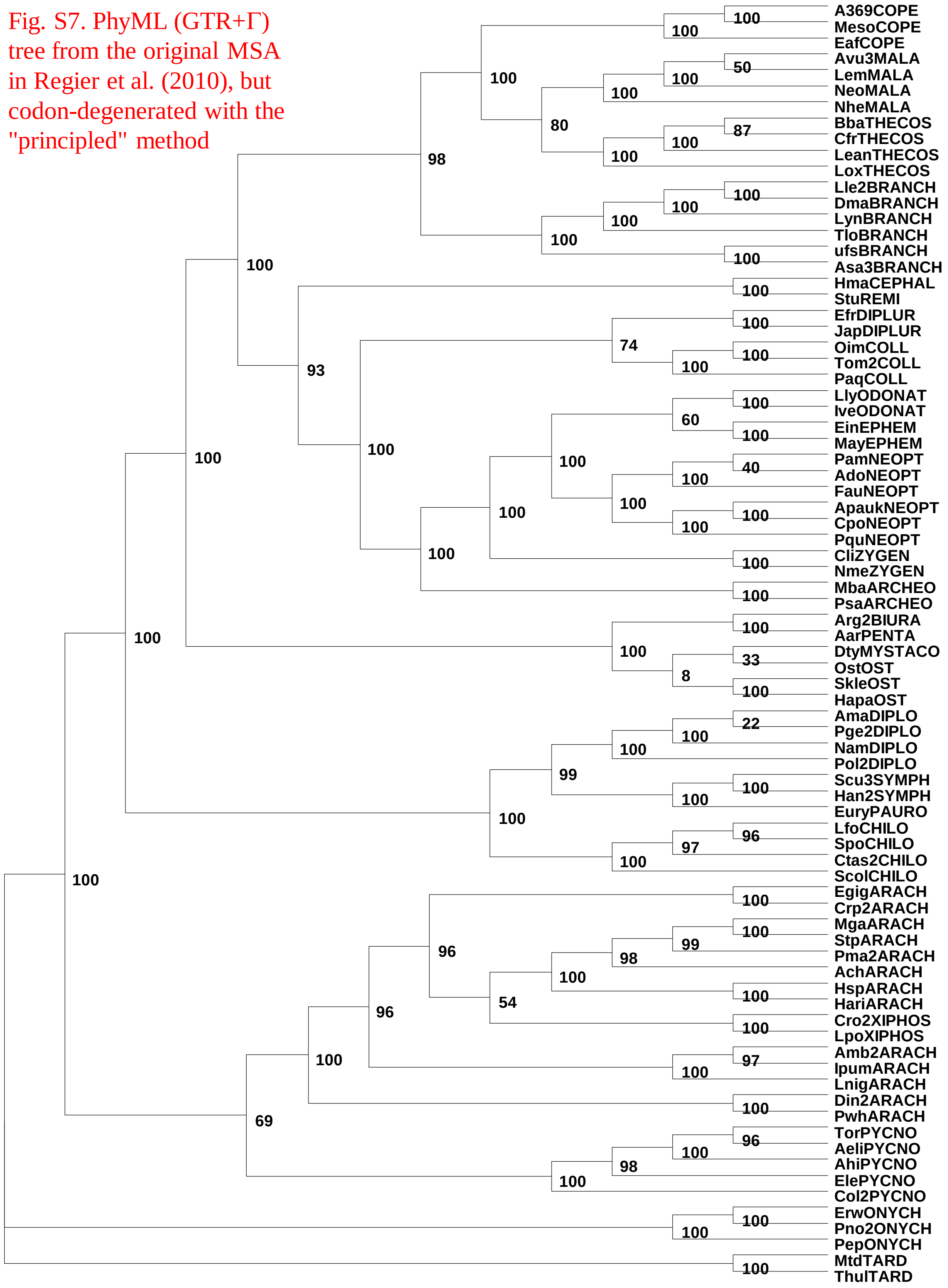

Fig. S8. PhyML (LG+Γ) tree from amino acid file translated from SuperMat.PHY.

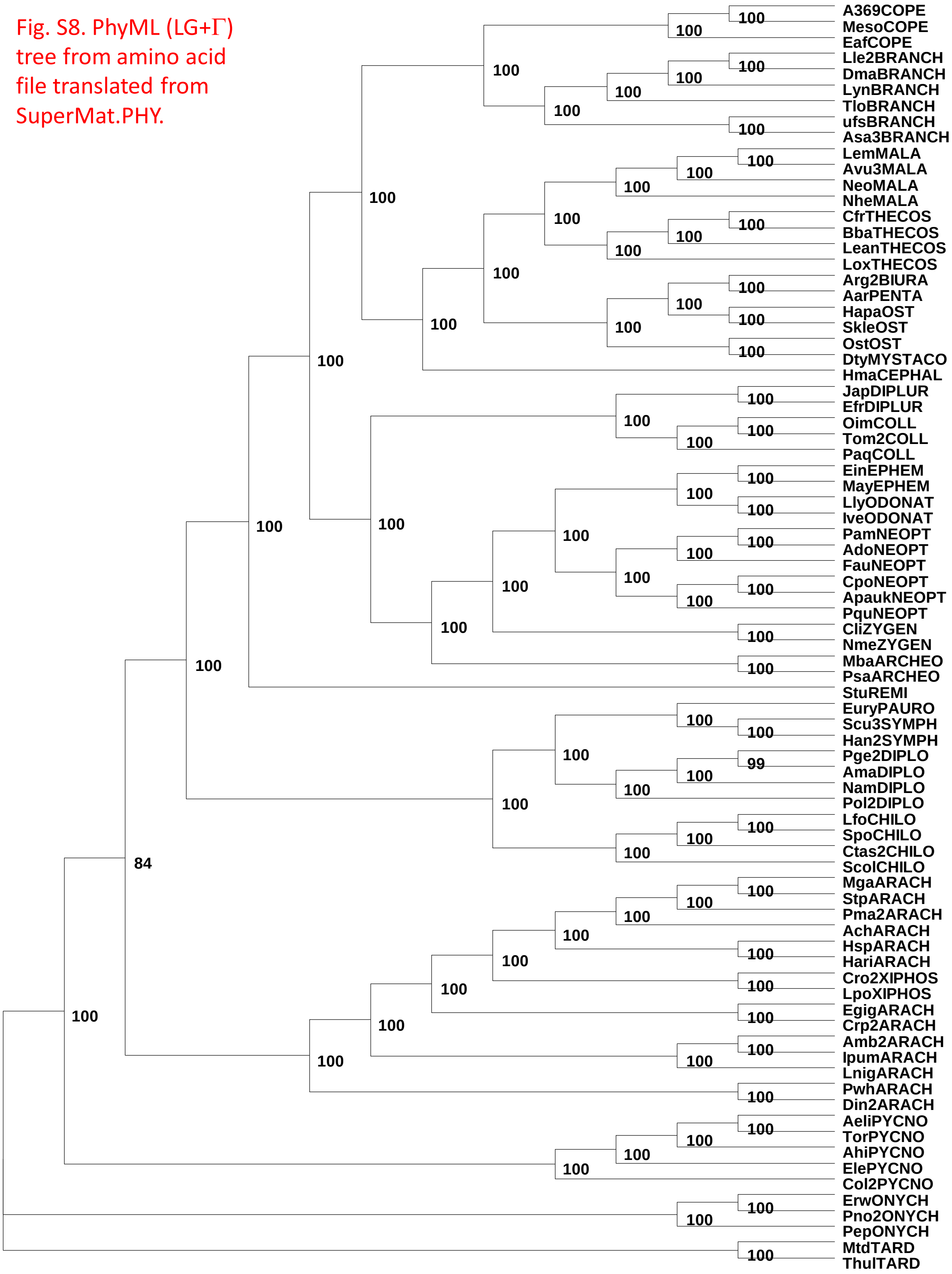

Fig. S9. PhyML3.1+(GTR+Γ)
Sites with both AGY and UCN (two serine codon subfamilies) are removed. Codon sequences are then degenerated in DAMBE

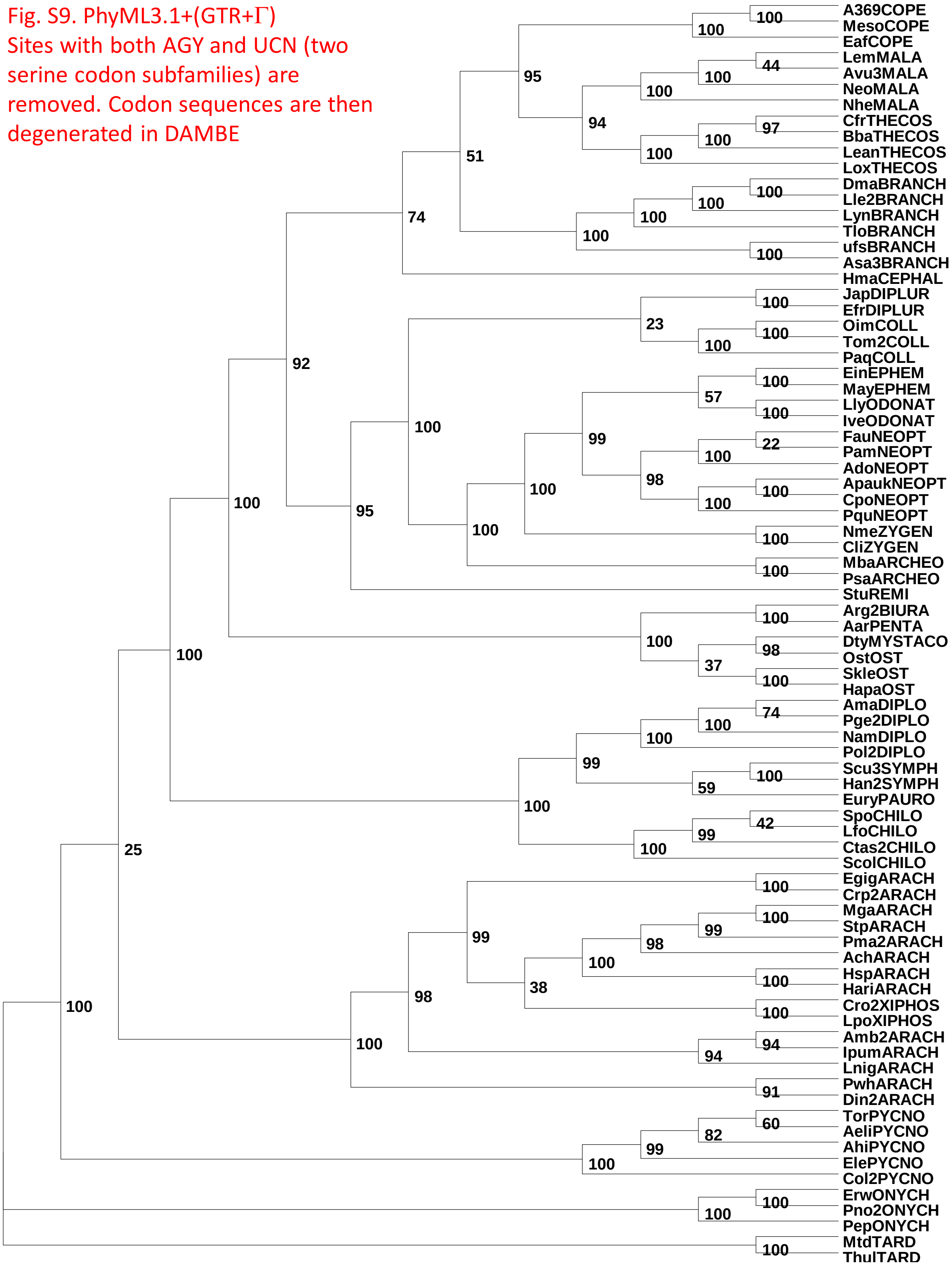

Appendix 1. Sequence names matched to genus name

Trees were originally shown with sequence names as in the sequence file SuperMatDegen.NEX. One reviewer suggests to show Fig. 5 with genus name, but other readers prefer the sequence names to facilitate comparison, hence this matching list between sequence names and genus name, recompiled from Regier et al.(2010).

OTU name	Genus	OTU name	Genus
FauNEOPT	Forficula	AchARACH	Aphonopelma
LfoCHILO	Lithobius	HapaOST	Harbansus
LpoXIPHOS	Limulus	LnigARACH	Leiobunum
MesoCOPE	Mesocyclops	LoxTHECOS	Loxothylacus
MgaARACH	Mastigoproctus	LynBRANCH	Lynceus
NamDIPLO	Narceus	SkleOST	Skogsbergia
NheMALA	Nebalia	AdoNEOPT	Acheta
OstOST	Cypridopsis	EfrDIPLUR	Eumesocampa
PaqCOLL	Podura	EinEPHEM	Ephemerella
StuREMI	Speleonectes	IveODONAT	Ischnura
ThulTARD	Thulinius	LlyODONAT	Libellula
TloBRANCH	Triops	MbaARCHEO	Machiloides
TorPYCNO	Tanystylum	NmeZYGEN	Nicoletia
ApaukNEOPT	Antheraea	AmaDIPLO	Abacion
CpoNEOPT	Cydia	Han2SYMPH	Hanseniella
PquNEOPT	Prodoxus	Pge2DIPLO	Polyzonium
A369COPE	Acanthocyclops	Pol2DIPLO	Polyxenus
Arg2BIURA	Argulus	Scu3SYMPH	Scutigerella
Asa3BRANCH	Artemia	Pno2ONYCH	Peripatoides
BbaTHECOS	Semibalanus	AeliPYCNO	Achelia
CfrTHECOS	Chthamalus	AhiPYCNO	Ammothea
CliZYGEN	Ctenolepisma	Amb2ARACH	Amblyomma
DmaBRANCH	Daphnia	Avu3MALA	Armadillidium
EafCOPE	Eurytemora	Col2PYCNO	Colossendeis
HariARACH	Hadrurus	Cro2XIPHOS	Carcinoscorpius
HmaCEPHAL	Hutchinsoniella	Crp2ARACH	Cryptocellus
HspARACH	Heterometrus	Ctas2CHILO	Craterostigmus
LeanTHECOS	Lepas	Din2ARACH	Dinothrombium
LemMALA	Libinia	DtyMYSTACO	Derocheilocaris
MayEPHEM	Hexagenia	EgigARACH	Eremocosta
JapDIPLUR	Metajapyx	ElePYCNO	Endeis
NeoMALA	Neogonodactylus	ErwONYCH	Euperipatoides
OimCOLL	Orchesella	EuryPAURO	Eurypauropus
PamNEOPT	Periplaneta	IpumARACH	Idiogaryops
PsaARCHEO	Pedetontus	Lle2BRANCH	Limnadia

ScolCHILO	Scutigera	MtdTARD	Milnesium
SpoCHILO	Scolopendra	PepONYCH	Peripatus
Tom2COLL	Tomocerus	Pma2ARACH	Phrynus
ufsBRANCH	Streptocephalus	PwhARACH	Prokoenenia
AarPENTA	Armillifer	StpARACH	Stenochrus

Althaus E, Caprara A, Lenhof HP, Reinert K. 2002. Multiple sequence alignment with arbitrary gap costs: computing an optimal solution using polyhedral combinatorics. Bioinformatics 18 Suppl 2:S4-S16.

Edgar RC. 2004a. MUSCLE: a multiple sequence alignment method with reduced time and space complexity. BMC Bioinformatics 5:113.

Edgar RC. 2004b. MUSCLE: multiple sequence alignment with high accuracy and high throughput. Nucleic Acids Res 32:1792-1797.

Gupta SK, Kececioglu JD, Schaffer AA. 1995. Improving the practical space and time efficiency of the shortest-paths approach to sum-of-pairs multiple sequence alignment. J Comput Biol 2:459-472.

Katoh K, Asimenos G, Toh H. 2009. Multiple alignment of DNA sequences with MAFFT. Methods Mol Biol 537:39-64.

Lipman DJ, Altschul SF, Kececioglu JD. 1989. A tool for multiple sequence alignment. Proceedings of the National Academy of Sciences of the United States of America 86:4412-4415.

Regier JC, Shultz JW, Zwick A, Hussey A, Ball B, Wetzer R, Martin JW, Cunningham CW. 2010. Arthropod relationships revealed by phylogenomic analysis of nuclear protein-coding sequences. Nature 463:1079-1083.

Reinert K, Stoye J, Will T. 2000. An iterative method for faster sum-of-pairs multiple sequence alignment. Bioinformatics 16:808-814.

Stoye J, Moulton V, Dress AW. 1997. DCA: an efficient implementation of the divide-and-conquer approach to simultaneous multiple sequence alignment. Comput Appl Biosci 13:625-626.

Thompson JD, Higgins DG, Gibson TJ. 1994. CLUSTAL W: improving the sensitivity of progressive multiple sequence alignment through sequence weighting, positions-specific gap penalties and weight matrix choice. Nucleic Acids Research 22:4673-4680.

Xia X. 2018. DAMBE7: New and improved tools for data analysis in molecular biology and evolution. Molecular Biology and Evolution 35:1550–1552.

Xia X. 2000. Data analysis in molecular biology and evolution. Boston: Kluwer Academic Publishers.
